# A Systematic Review and Bayesian Network Meta-Analysis on the Effect of Different Anticoagulants on the Prophylaxis of Post-Thrombotic Syndrome after Deep Venous Thrombosis

**DOI:** 10.3390/jcm12237450

**Published:** 2023-11-30

**Authors:** Jingbo Shao, Qianwen Zhou, Fukang Jin, Christoph Reissfelder, Martin Sigl, Vugar Yagublu, Michael Keese

**Affiliations:** 1 Department of Surgery, Universitätsmedizin Mannheim, Medical Faculty Mannheim, Heidelberg University, 68167 Mannheim, Germany; jingbo.shao@medma.uni-heidelberg.de (J.S.); qianwen.zhou@medma.uni-heidelberg.de (Q.Z.); fukang.jin@medma.uni-heidelberg.de (F.J.); christoph.reissfelder@medma.uni-heidelberg.de (C.R.); vugar.yagublu@umm.de (V.Y.); 2 European Center of Angioscience ECAS, Medical Faculty Mannheim, Heidelberg University, 68167 Mannheim, Germany; martin.sigl@umm.de; 3 Department of Cardiology, Angiology, Haemostaseology and Medical Intensive Care, Universitätsmedizin Mannheim, Medical Faculty Mannheim, Heidelberg University, 68167 Mannheim, Germany; 4 Department of Vascular Surgery, Theresienkrankenhaus, 68165 Mannheim, Germany

**Keywords:** postthrombotic syndrome, anticoagulants, Villalta score, Bayesian network meta-analysis

## Abstract

Background: Postthrombotic syndrome (PTS) has a major impact on the quality of life after deep venous thrombosis (DVT). From clinical practice and related trials, anticoagulants show potential for reducing the occurrence and alleviating the symptoms of PTS. Methods: A systematic review and Bayesian network meta-analysis (NMA) were conducted by combing the literature from the databases of MEDLINE, Embase, Web of Science, Cochrane Libraries, and ClinicalTrials, through a variety of medical subject headings (Mesh) and PTS keywords. With regard to PTS prophylaxis, all anticoagulant-related randomized controlled trials (RCTs) and observational studies were assessed. The network model was conducted through the R software, and further comparisons were conducted using the Bayesian hierarchical random effects model. The odds ratio (OR) and the corresponding 95% CI were calculated for analysis. Results: Data from two RCTs and nine non-randomized studies meeting the selection criteria were included in the Bayesian analysis model, which incorporated seven anticoagulants. Edoxaban (OR: 0.42, 95% CI: 0.18–1.0) and rivaroxaban (OR: 0.54, 95% CI: 0.38–0.76) were significantly more effective than warfarin in the prevention of PTS (Villalta score ≥ 5). A subgroup analysis based on the severity of PTS showed that rivaroxaban was more effective than warfarin, with OR: 0.59, 95% CI: 0.41–0.84 (Villalta score 5 to 14) and OR: 0.48, 95% CI: 0.22–0.9 (Villalta score ≥ 15, ulceration), respectively. Edoxaban had the highest probability (80.1%) of providing preventive benefits for PTS. For mild/moderate and severe PTS, rivaroxaban provided the highest benefits in preventing PTS (89.3% and 85.6%, respectively). Conclusion: Edoxaban demonstrated a better prophylactic effect on PTS (Villalta score > 5), while rivaroxaban displayed a better effect against mild/moderate (Villalta score 5 to 14) and severe PTS (Villalta score ≥ 15, ulceration).

## 1. Introduction

Postthrombotic syndrome (PTS) is a widely prevalent and chronic sequela following lower-extremity deep venous thrombosis (DVT) [1]. Approximately 20% to 50% of all DVT patients develop PTS [2,3]. PTS leads to a substantial impairment in the quality of life [4]. In the United States, the diagnosis of PTS leads to an increase in total medical costs of 20% compared to non-PTS patients during the 12-month follow-up [5].

Due to multiple physiopathologic changes during PTS, including venous hypertension and a cascade of inflammatory responses, symptoms/signs of PTS may also vary among patients. Therefore, there is no gold standard in diagnosing PTS [6]. The common pathophysiology of secondary venous insufficiency and recurrent DVT typically manifests itself clinically with chronic pain, edema, swelling, heaviness, fatigue, itchiness, the formation of varicose veins, hyperpigmentation, eczema, and ulceration [7,8]. Moreover, symptoms of PTS tend to exacerbate after exercise, while rest and leg elevation alleviate the discomfort. Generally, after DVT events, the abovementioned symptoms develop between three and six months [9], and, in some cases, symptoms can occur up to two years afterwards [2,4].

There are several clinical tests or scales proposed in the diagnosis and definition of PTS, including the venous clinical severity score (VCSS) [10], the clinical-etiological-anatomical-pathophysiological (CEAP) classification [11], the Widmer scale [12], Brandjes [13], Ginsberg [14], and the Villalta scale [15]. Unlike VCSS, all other scales or scores are specific in determining the degree of PTS [16]. The International Society on Thrombosis and Haemostasis (ISTH) recommends the Villalta scale as the most appropriate method for assessing the severity of PTS [17].

The Villalta scale takes not only objective signs but also subjective symptoms into consideration, and demonstrates good validity, inter-rater reliability, and the capacity for wide applications in clinical trials [18]. The components of the scale are five patient-reported symptoms (pain, cramps, heaviness, paresthesia, pruritus) and six clinician-assessed indicators (pretibial edema, skin induration, hyperpigmentation, redness, venous ectasia, pain on calf compression). Each component is graded using four points (from 0 to 3), and the points are added to generate a total score. A score of < 5 excludes PTS, between 5 and 9 indicates mild PTS, between 10 and 14 indicates moderate PTS, and a score ≥ 15 or ulceration indicates severe PTS.

Since the corresponding manifestations of PTS can severely damage the life quality of patients, the prophylaxis of PTS after DVT is essential for any patient. Though endovascular therapies, such as systemic thrombolysis, catheter-directed thrombolysis, and pharmacomechanical thrombolysis, have been used for the prevention of PTS [19,20], adverse events like bleeding have occurred [21]. Therefore, the European Society for Vascular Surgery recommended these techniques in their guideline in 2021 for selected patients only [22]. Alternatively, there is still controversy on the prophylactic effect of anticoagulants for PTS. Therefore, the American Heart Association (AHA) has suggested that there is a need to optimize the intensity and duration of anticoagulants, and further studies are necessary to compare the effects of different anticoagulants on PTS [23].

Therefore, to provide a better understanding of this issue, we here conducted a systematic review and a Bayesian NMA to compare seven anticoagulants in terms of their prevention of PTS.

## 2. Methods

This study, utilizing NMA, was arranged and conducted according to the Preferred Reporting Items for Systematic Reviews and Meta-Analyses (PRISMA) statement extension for NMA [24], with a checklist in Table S1 and flow diagram in Figure S1. Its protocol was registered on PROSPERO with ID: CRD42022357925.

### 2.1. Search Strategy

A comprehensive survey of multiple databases (MEDLINE, Embase, Web of Science, Cochrane Libraries, and ClinicalTrials) for research dating from January 1990 to July 2022 was conducted using a variety of keywords associated with concepts relating to PTS. Qualitative search terms containing population, intervention, control, and outcomes (PICO) were utilized as a search strategy. Text words with a truncation symbol and terms with MeSH—such as “postthrombotic syndrome”, “postphlebitic syndrome”, “chronic vein insufficiency”, “anticoagulants”, and “direct oral anticoagul *”—were used. Additionally, reference lists of all relevant meta-analyses and papers pertaining to potential data sources were manually examined to ensure a comprehensive search.

### 2.2. Selection Criteria

We conducted a Bayesian NMA including RCTs and observational studies. Studies that met the following criteria were included: (1) a confirmed diagnosis of DVT through at least one of these examinations: clinical signs, symptoms, laboratory tests, venography, and ultrasonography; (2) at least a two-arm clinical trial involving no fewer than two anticoagulants; (3) a clear description of the Villalta scale and the severity of PTS; and (4) studies reporting the incidence of PTS, such as a hazard ratio (HR), odds ratio (OR), or 95% confidence interval (95% CI). Alternatively, studies were included if the calculation of these values was possible. Studies that have one of the following characteristics were excluded: (1) irrelevant population to PTS, (2) a comparison of therapies without a focus on anticoagulants, (3) reviews and studies without adequate data on the incidence of PTS, or (4) the corresponding full text was not written in English.

The primary outcome was the event or incidence of PTS among patients who took anticoagulants. A predefined subgroup analysis according to the PTS’s severity and DVT recurrence was then conducted. The incidence of mild/moderate PTS (Villalta score 5 to 14), severe PTS (Villalta score over 14 or ulcer), and recurrent DVT was considered the secondary outcome.

### 2.3. Data Extraction and Quality Assessment

Two reviewers (J.S. and Q.Z.) independently screened all the potentially eligible studies. Then, the available data, such as patient population, age, study type, therapeutic schedule, duration of follow-up, and outcomes of interest, were extracted. Each reviewer created an electronic database, and any disagreements on data extraction and evaluation were resolved through discussion. A third reviewer (F.J.) was in charge of the consultation when discrepancies arose after discussion.

The quality of the observational cohort and cross-sectional studies was individually assessed by two reviewers (J.S. and Q.Z.). For observational studies, the entire assessment process was conducted in accordance with the Risk of Bias in Non-randomized Studies of Interventions (ROBINS-I)’s standard seven aspects: confounding, selection of patients, classification of interventions, deviation from intended interventions, missing data, measurement of outcomes, and selection of the reported result. After assessment, each reviewer rated the quality of the studies and made an overall risk-of-bias determination.

Regarding the RCTs, we applied version 2 of the Cochrane risk-of-bias tool for randomized trials (RoB 2) to conduct an overall assessment of the potential bias across five domains: the randomization process, deviation from intended interventions, missing outcome data, measurement of the outcome, and selection of the reported result. An overall risk of bias for the RCTs was determined based on these five domains.

### 2.4. Statistical Analysis

#### 2.4.1. Bayesian Network Model

In our study, we systematically constructed a Bayesian network model and performed an NMA using the R software (version 4.1.0, R Core Team, Vienna, Austria). Furthermore, with aid from the R packages—rjags (version 4.3.0), coda (version 0.19-4), R2OpenBUGS (version 3.2-3.2.1), metafor (version 3.4-0) and gemtc (version 1.0-1)—we specified and compiled Bayesian hierarchical models. Random effect models were utilized, and the OR and the corresponding 95% CI were calculated as indicators since the abstracted data were binary outcome variables. Afterward, heterogeneity for the given network model was analyzed according to three model types: unrelated study effects, unrelated mean effects, and consistency. Meanwhile, a global I^2^ statistical analysis was performed, which acted as an indicator of heterogeneity. Heterogeneity is considered low, moderate, or high when the value reaches 25%, 50%, and 75%, respectively.

The Bayesian NMA model in our study considered both direct and indirect comparisons of two anticoagulants and provided a comprehensive and hierarchical ranking. The posterior distributions were determined via Markov chain Monte Carlo (MCMC) sampling with 5000 burn-ins and 100,000 iterations using our chains. Gelman–Rubin plots were made for the sake of assessment of the coverage. The potential scale reduction factor (PSRF) was used to determine the optimal setting by a comparison of the within-chain and between-chain variance, where the PSRF should be less than 1.05 to indicate an approximate convergence [25]. Additionally, in terms of anticoagulants against PTS, the probability of rank was calculated by comparing each anticoagulant’s OR to that of all the other anticoagulants. The data of this Bayesian NMA were loaded into the dmetar package (version 0.0.9000), which generated data for the surface under the cumulative ranking (SUCRA). This index, ranging from 0 to 1, indicated which anticoagulant was better in terms of providing a prophylactic effect for PTS. Finally, the corresponding SUCRA data were supplied to a function derived from Veroniki et al. [26] to create a rank-heat plot and show the overall preventive effect of anticoagulants against PTS.

#### 2.4.2. Inconsistency Analysis

According to the description from Dias et al. [27], only when those comparisons are parts of a closed loop can an inconsistency analysis be considered to further evaluate the network inconsistency between indirect and direct comparisons. In this study, our inconsistency analysis was established by a loop consisting of rivaroxaban, dabigatran, and warfarin.

#### 2.4.3. Sensitivity Analysis and Meta-Regression

As a sensitivity analysis can sometimes reveal heterogeneity, one was conducted to examine the reliability of this Bayesian NMA model. All the included trials were divided based on their quality assessment and sample size to check if these two factors could influence the reliability of the Bayesian framework; a comparison of the deviance information criterion (DIC) of the two Bayesian NMA models was used, where a lower DIC value indicates a better-fitted model.

Meta-regression is used to assess if a specific feature has an impact on a network model. It is a useful tool in identifying the cause of inconsistency and heterogeneity. Whether it should be performed or not is influenced by the evaluation of inconsistency and heterogeneity. If the statistical analysis using the gemtc package in R displays significant heterogeneity and inconsistency, a meta-regression would be performed with the built-in function in the gemtc package to identify which covariate has an impact on the effect estimate.

#### 2.4.4. Publication Bias

In this NMA model, a comparison-adjusted funnel plot was applied for small studies. Through the comparison of the distribution of two treatments in the plot, publication bias was visualized. Finally, Egger’s test was conducted for further statistical analysis. The asymmetrical distribution of points and a *p* < 0.05 in Egger’s test indicated publication bias.

## 3. Results

### 3.1. Study Selection and Quality Assessment

**Figure 1:**
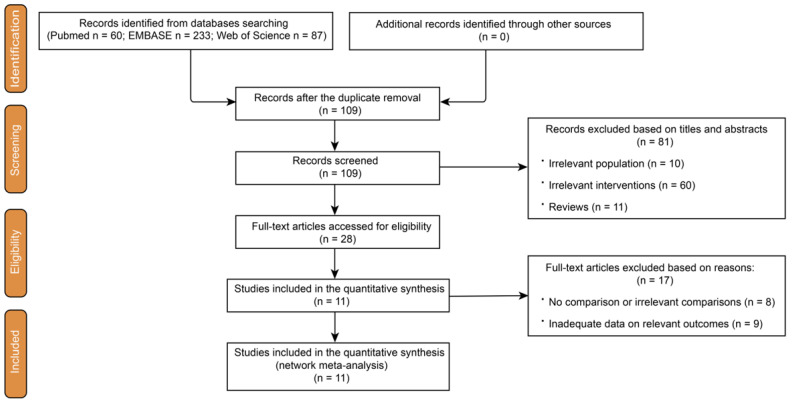
Flow diagram for the study selection based on PRISMA.

**Figure 2:**
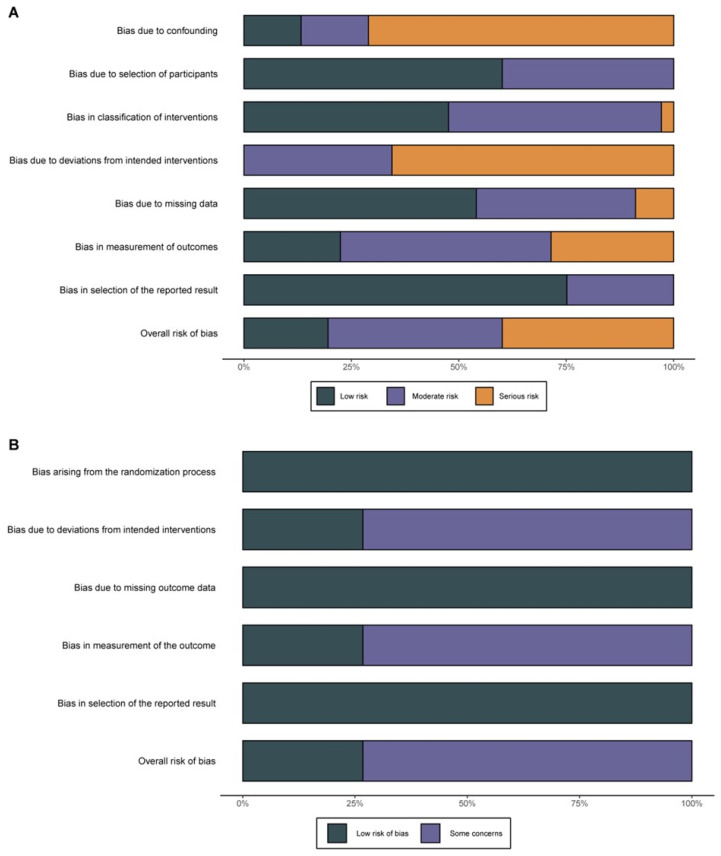
Summary of risks of bias. (**A**) Non-randomized studies according to ROBINS-I and (**B**) RCTs according to RoB 2. (ROBINS-I: Risk of Bias in Non-randomized Studies of Interventions; RoB 2: Version 2 of the Cochrane risk-of-bias tool for randomized trials).

After a comprehensive scouring of the electronic databases, a total of 380 records were identified. Additionally, after manually searching conference abstracts and relevant reference lists, no extra records were identified. A total of 271 records were removed due to duplication, and 109 trials were retained for further assessment. Subsequently, 70 trials were excluded due to an irrelevant population or intervention. Of the remaining 39 articles, 11 were reviews, which were also excluded. Afterward, the eligibility of the 28 relevant articles was finally determined through full-text evaluations, wherein 11 articles that met the inclusion criteria were incorporated into our Bayesian NMA [28,29,30,31,32,33,34,35,36,37,38] (Figure 1). Of these chosen clinical trials, nine trials were non-randomized studies, and the other two included trials were RCTs. Regarding all the included non-randomized studies, two studies were rated as “high risk”, another five were “moderate risk”, and the last two studies were “low risk” for bias. A potential high risk found was by Jeraj et al. [31], who described the treatments and the assessment of symptoms. The other one had a significant difference in patients’ characteristics between groups, especially in the group of novel direct oral anticoagulants (DOAC) [33]. The process of quality rating was conducted according to ROBINS-I (Figure 2A). After rating the remaining two RCTs, one study [35] was considered to have some concerns according to ROB 2, and the other [36] one was low risk (Figure 2B).

### 3.2. Characteristics and Baseline Data of the Studies Included in the NMA

With regard to the abovementioned 11 clinical trials that met the criteria for the NMA, all the related characteristics of the 11 included studies are summarized in Table S2. Of the non-randomized studies, four out of nine were prospective cohort studies, while four were cross-sectional studies, and only one was a post hoc analysis. In terms of the types of clinical trials, 10 out of 11 trials were double-arm trials. The majority of the included double-arm trials focused on a comparison between rivaroxaban and warfarin (seven trials). Among the remaining double-arm trials, one examined the comparison between low-molecular-weight heparin (LMWH) and LMWH + rosuvastatin, while another one explored the relationship between dabigatran and warfarin. The other trial investigated the preventive effect of enoxaparin and coumarin. Only one multi-arm clinical trial was included, which focused on DOACs (edoxaban, dabigatran, rivaroxaban, etc.). The duration of the follow-up period ranged from three months to five years. The shortest follow-up time was three months, as reported by Norberto et al. [35]. On the other hand, González-Fajardo et al. [29] had the longest surveillance and follow-up, which lasted five years. In Table S1, all the data were recorded as numbers of events from groups determined via the Villalta scale. Subsequently, data on recurrent DVT were also listed. An overview of the summarized data on sample size, age, gender, body mass index (BMI), use of elastic stockings, and proximal and unprovoked DVT, is shown in Table S3. The included clinical trials comprised 3812 patients, consisting of 1965 males and 1847 females. A disproportion of gender was only found in the trial by Sebastian et al. [34]. The majority of patients who met the criteria of our NMA were diagnosed with proximal DVT (78.1%), and around half of these patients were found to have unprovoked DVT (47.1%). Table S3 presents an overview of the baseline data from all included studies.

### 3.3. Bayesian Network Meta-Analysis

**Figure 3:**
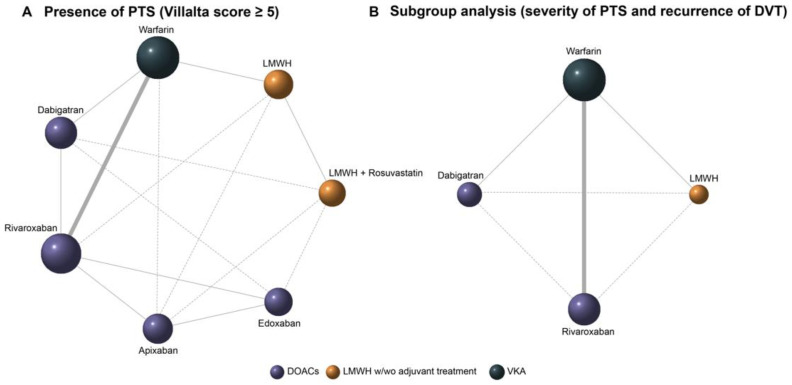
The network analysis plots. (**A**) Presence of PTS with a Villalta score ≥ 5. (**B**) Subgroup analysis: severity of PTS based on Villalta score and recurrence of DVT. (PTS: postthrombotic syndrome; DVT: deep venous thrombosis).

First of all, based on the 11 included trials, which were previously identified for further analysis, network plots were drawn to present the relationships of all these anticoagulants. Figure 3A shows an overall comparison with seven nodes connected by eight solid lines and seven dashed lines. The size of the nodes corresponds to the sample sizes of each anticoagulant, and the thickness of the solid line segments represents the number of studies included for the paired anticoagulants. The Bayesian NMA model generated direct and indirect comparisons between the involved anticoagulants. In Figure 3, the solid lines represent direct comparisons in the Bayesian NMA model. The dashed lines, on the other hand, represent indirect comparisons. Moreover, the anticoagulants were classified into three categories: DOACs, LMWH w/wo adjuvant treatment, and VKA (vitamin K antagonist). Subgroup analyses were performed based on the abstracted data according to the severity of PTS and DVT recurrence. As listed in Table S2, the example sizes and structures of the two subgroups were the same. Therefore, one network plot in Figure 3B describes the relationships in the subgroup analysis. Each subgroup analysis consists of three direct and three indirect comparisons.

As an important part of the Bayesian NMA, the process of MCMC simulations created the convergence of the algorithm to determine which settings are optimal. In Figure S2, trace plots (left) are provided to show the model convergence, demonstrating that the model converges well. Throughout the density plots (right), the curves tended to be regularly distributed. In our study, an assessment of convergence via the Gel-Rubin plot (Figure S3) was also performed to demonstrate the potential scale reduction factor (PSRF), which compared the variance within each chain to the variation between chains and the evolution of both variances over time. In Figure S3, the PSRF gradually approaches 0 with increasing iterations. Finally, it reaches 1.002 after 1 × 10^5^ iterations.

### 3.4. Assessment of Heterogeneity and Inconsistency

The assessments of heterogeneity were carried out using the mtc.anohe function in the gemtc R package. Figure S4 shows the overall studies for anticoagulants with a Villalta score ≥ 5. The global I^2^ of all of the 11 included studies was 2%. Except for the comparison of anticoagulants (warfarin vs. dabigatran, I^2^ = 66.7%), there was no moderate-to-high (I^2^ ≥ 50%) pooled heterogeneity among any comparisons. The pooled heterogeneity of the study comparing rivaroxaban and dabigatran was close to the level of moderate (I^2^ = 44%). The sample sizes of all subgroups were the same, containing nine studies and 3582 cases.

As shown in Figure S5, the global I^2^ of the subgroup (14 ≥ Villalta score ≥ 5 and without ulceration) was 11%. The heterogeneity was mostly derived from a comparison between the anticoagulants warfarin and rivaroxaban (I^2^ = 12.8%). Another subgroup analysis contains all the studies of severe PTS (Villalta score ≥ 15 or ulceration), and the heterogeneity among all these studies was low (I^2^ = 0%) (Figure S6). However, in the subgroup of recurrent DVT, the global I^2^ was 82.9%, indicating high heterogeneity (Figure S7).

**Figure 4:**
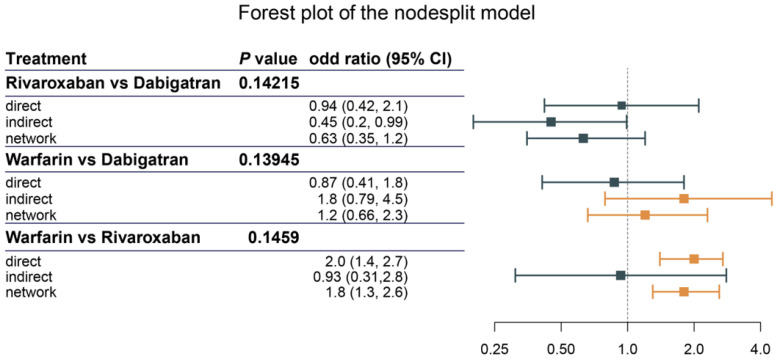
The forest plot of the nodesplit model, including the anticoagulants in the closed loop. *p*-value < 0.05 indicated inconsistency in the network.

Regarding inconsistency, a node-splitting analysis was conducted to determine the inconsistency of the NMA. In Figure 4, both direct and indirect evidence was calculated to show the inconsistency in the closed loop consisting of rivaroxaban, dabigatran, and rivaroxaban. Direct evidence was presented as ORs of rivaroxaban and dabigatran (OR: 0.94, 95% CI: 0.42–2.1), warfarin and dabigatran (OR: 0.87, 95% CI: 0.41–1.8), and warfarin and rivaroxaban (OR: 2.0, 95% CI: 1.4–2.7). The corresponding indirect values were 0.45 (95% CI: 0.2–0.99), 1.8 (95% CI: 0.79–4.5), and 0.93 (95% CI: 0.31, 2.8), respectively. All available evidence (network) was 0.63 (95% CI: 0.35–1.2) in rivaroxaban vs. dabigatran, 1.2 (95% CI: 0.66–2.3) in warfarin vs. dabigatran, and 1.8 (95% CI: 1.3–2.6) in warfarin vs. rivaroxaban. The *p*-values were 0.142, 0.139, and 0.146, respectively. These indicated that there was no inconsistency in our network model.

### 3.5. Pairwise Comparison of Anticoagulants

**Figure 5:**
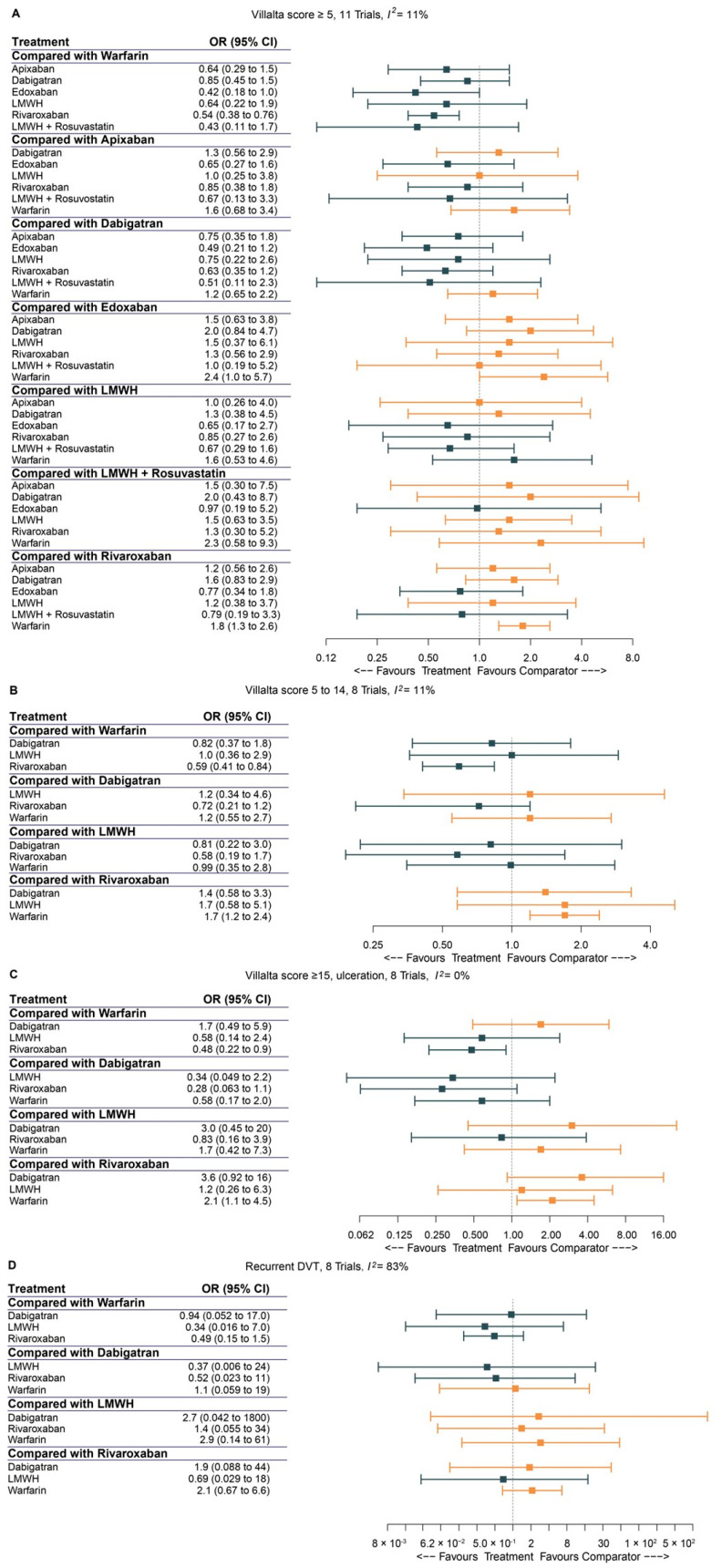
The forest plots for pairwise comparisons between different anticoagulants. (**A**) Villalta score ≥ 5; (**B**) subgroup: mild/moderate PTS, Villalta 5 to 14; (**C**) subgroup: severe PTS, Villalta score ≥ 15, ulceration; (**D**) subgroup: recurrent DVT. (PTS: postthrombotic syndrome; DVT: deep venous thrombosis; LMWH: low-molecular-weight heparin; OR: odds ratio; CI: confidence interval).

Figure 5A depicted the pairwise comparisons in one network model. In Figure 5B–D, pairwise comparisons of the three subgroups under different conditions (mild/moderate PTS, severe PTS, and recurrent DVT) were also performed. In Figure 5A, the forest plot demonstrated that edoxaban (OR: 0.42, 95% CI: 0.18–1.0) and rivaroxaban (OR: 0.54, 95% CI: 0.38–0.76) were both significantly more effective than warfarin in the prevention of PTS (Villalta score ≥ 5), compared to apixaban (OR: 0.64, 95% CI: 0.29–1.5); dabigatran (OR: 0.85, 95% CI: 0.45–1.5); LMWH (OR: 0.64, 95% CI: 0.22–1.9); and LMWH + rosuvastatin (OR: 0.43, 95% CI: 0.11–1.7). In a subgroup of mild/moderate PTS (Figure 5B) and severe PTS (Figure 5C), only rivaroxaban resulted in a significantly reduced occurrence of PTS compared to the usage of warfarin (OR: 0.59, 95% CI: 0.41–0.84 and OR: 0.48, 95% CI: 0.22–0.9, respectively).

Regarding the recurrence of DVT, the pairwise comparisons of anticoagulants did not prove that there were any superior drugs among warfarin, dabigatran, LMWH, and rivaroxaban (Figure 5D).

Accordingly, the relative effects of the included pairs were presented in summary tables with a log odds ratio in Table S4.

### 3.6. Ranking Probability and SUCRA

**Figure 6:**
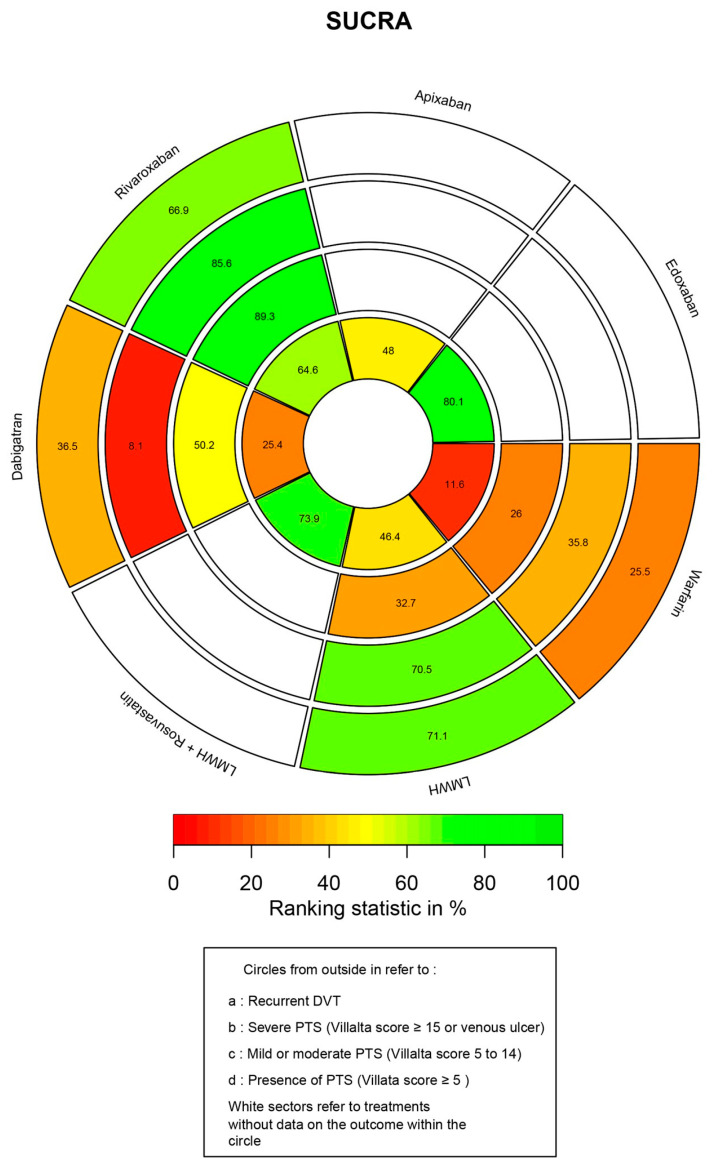
Rank-heat plot of seven anticoagulants on overall PTS, severity of PTS, and recurrence of DVT. The hue of each sector corresponds to the SUCRA value of the respective treatment and outcome, ranging from red (0%) to green (100%). The absence of color indicates that the underlying therapy was not included in the NMA in terms of outcomes. (PTS: postthrombotic syndrome; DVT: deep venous thrombosis; SUCRA: surface under the cumulative ranking).

All the rank probabilities were calculated, and the SUCRA values are shown in a rank-heat plot. In Figure 6, the area of each concentric circle is split into the seven anticoagulants. Each sector is colored based on the respective SUCRA; the scale ranges from the lowest (0%) to the greatest (100%) value.

In Figure 6, starting from the outermost concentric circle and moving towards the center, the circles represent the following categories: recurrent DVT, severe PTS, mild/moderate PTS, and the presence of PTS. Among all the anticoagulants, endoxaban had the highest SUCRA value (80.1%) for the prevention of PTS based on its Villalta score, followed by LMWH + rosuvastatin (73.9%), rivaroxaban (64.4%), apixaban (48.0%), LMWH (46.4%), and dabigatran (25.4%). As a traditional anticoagulant, warfarin ranked the worst against the occurrence of PTS (11.6%).

In terms of mild/moderate and severe PTS, rivaroxaban ranked the highest in the prevention of PTS with a SUCRA of 89.3% and 85.6%, respectively. While warfarin remained the lowest-ranked among the four anticoagulants in the group of mild/moderate PTS (26.0%). Unexpectedly, dabigatran had the lowest likelihood (8.1%) of preventing severe PTS events.

Concerning the recurrence of DVT, LMWH had a high probability of being superior to the other three anticoagulants (SUCRA: 71.1%), with a slight advantage in its SUCRA compared to rivaroxaban (SUCRA: 66.9%). Warfarin still ranked the lowest among the four anticoagulants (25.5%).

### 3.7. Publication Bias

**Figure 7:**
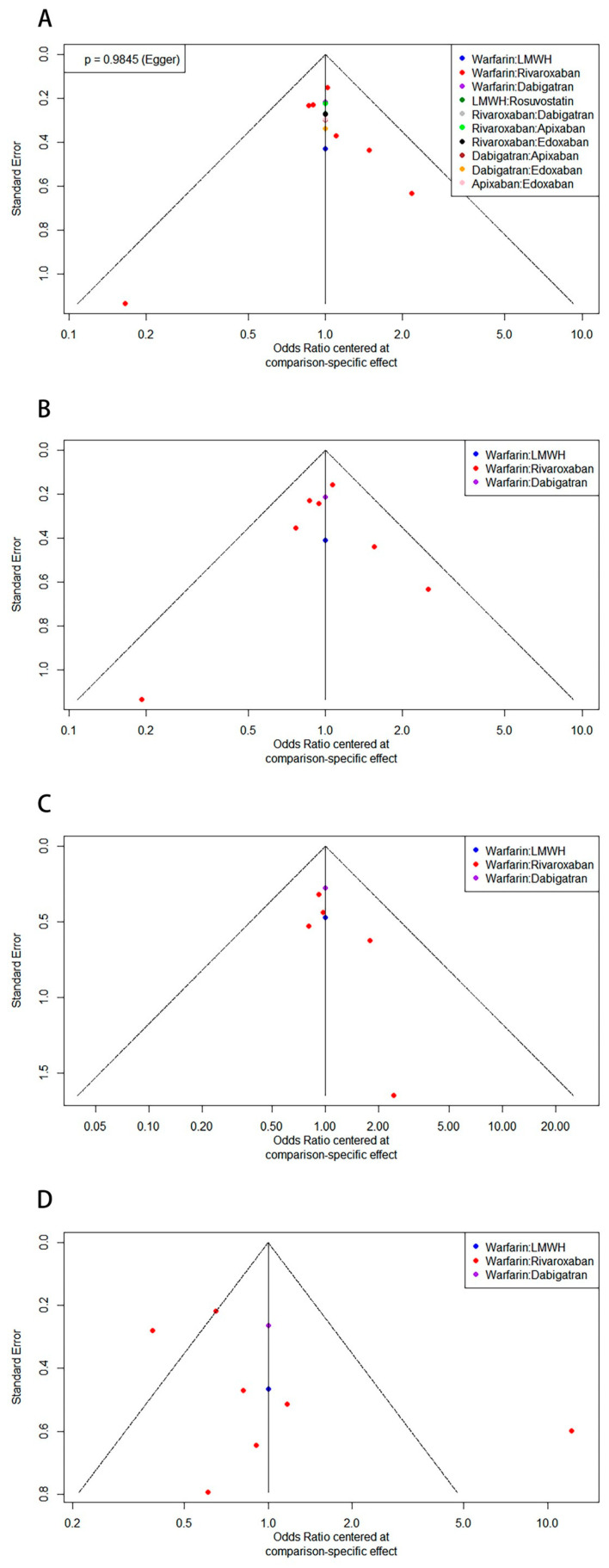
Comparison-adjusted funnel plots indicating publication bias: (**A**) all the studies met the selection criteria with a Villalta score ≥ 5; (**B**) subgroup: mild/moderate PTS (Villalta score 5 to 14); (**C**) subgroup: severe PTS (Villalta score ≥ 15, or venous ulcer); and (**D**) subgroup: recurrent DVT. (PTS: postthrombotic syndrome; DVT: deep venous thrombosis).

The publication bias of all the included studies was assessed using a comparison-adjusted funnel plot. As shown in Figure 7A, the comparison-adjusted funnel plot indicated a symmetrical distribution of estimates for all comparisons: the Egger’s test (*p* = 0.9845) indicated that there was no publication bias. Comparison-adjusted funnel plots for the subgroups are presented in Figure 7B–D. However, due to the small number of studies in each subgroup, Egger’s test may not have had adequate statistical power to detect publication biases in these subgroups.

### 3.8. Sensitivity Analysis

A sensitivity analysis was performed to assess the robustness of the Bayesian NMA model by evaluating studies with additional eligibility. The sensitivity analysis was conducted after excluding an RCT due to some concerns about its quality and two non-randomized studies with low methodological quality. As shown in Table S5, the rank order based on their SUCRAs was similar to the main analysis (Villalta score ≥ 5) after removing these studies with potentially high risks.

Likewise, after excluding small sample size studies (with a sample size of an individual group < 50), the SUCRA ranking was comparable with the results shown in the main analysis, indicating the robustness of our model.

The pD value describes the complexity of the NMA model. The complexity of the models was reduced from a pD = 19.8 to 15.5 in the first sensitivity analysis, and to 17.2 in the second sensitivity analysis.

The DIC is an indicator of model fit. In our sensitivity analysis, the DIC decreased from 45.5 (main analysis) to 35 (the first sensitivity analysis) and 36.8 (the second sensitivity analysis), respectively.

## 4. Discussion

To our knowledge, this is the first NMA comparing the influence of multiple anticoagulants that are commonly given to patients with DVT on PTS. In our study, comparisons between six monotherapies of anticoagulants (warfarin, apixaban, dabigatran, edoxaban, LMWH, and rivaroxaban) and one combination therapy (LMWH + rosuvastatin) were processed. Our NMA revealed the following findings: (i) In terms of the occurrence of PTS assessed via the Villalta scale, edoxaban produced more favorable outcomes compared to the other anticoagulants. (ii) In a subgroup analysis based on the severity of PTS, rivaroxaban was a more effective prophylaxis for mild/moderate and severe PTS. (iii) Edoxaban and rivaroxaban provided a significant improvement in the prevention of PTS (Villalta score > 5).

Our findings focused on the pharmacological options instead of interventional options, since, with the use of anticoagulants, adverse events, such as bleeding, are also possible, but less likely to occur. However, both treatment options originally focusing on the prevention of DVT recurrence can effectively function as PTS prevention [39]. Mechanical obstruction after a serial response of thrombosis-related inflammation and thrombosis cause venous hypertension and valvular reflux, which plays an essential role in PTS pathophysiology [40]. Pharmacologic and mechanical prophylaxis prevent or reduce the development of a thrombus burden and promote an earlier recanalization of the obstructed vessel [41,42].

In terms of interventional therapies, many endovascular therapies were introduced, and related randomized controlled trials have already been performed. To compare the long-term therapeutic effects between catheter-directed thrombolysis (CDT) plus standard anticoagulants and anticoagulants alone, the CaVenT study [43] recruited 209 patients with proximal DVT and compared CDT plus anticoagulants to standard anticoagulants alone. The difference in the absolute risk of PTS was 28% for a five-year follow-up and 14.4% for a 24-month follow-up, indicating that clinical benefits were obtained after adding CDT [20]. On the other hand, the ATTRACT trial [21] has proven that there is no significant difference in the proportion of PTS in a group receiving an anticoagulation treatment with pharmacomechanical thrombolysis and in a control group of anticoagulants. However, the severity of the PTS was lower in the interventional group. Here, the Villalta scale showed that patients with moderate or severe PTS seemed to benefit from pharmacomechanical thrombolysis. Recently, an update from the CAVA trial suggested that additional ultrasound-accelerated CDT was superior to the standard treatment (anticoagulant therapy, compression therapy, and early ambulation) in the prevention of PTS [19]. However, the adverse effects of CDT (most often bleeding) should not be ignored [44].

An anticoagulant treatment is primarily indicated to prevent the occurrence of embolic events or DVT recurrence [8]. However, thrombus regression is also supported, which is slow after the initial three months before, gradually, the residual venous obstruction (RVO) becomes stabilized, which is correlated with the dysfunction of venous valves and vein wall fibrosis [45]. During this period, sterile inflammatory processes are activated. Many inflammatory markers (CRP, IL-6, IL8) and adhesion molecules (P-selectin, ICAM-1, VCAM-1, etc.) are released, causing pathophysiologic cascades [46,47,48]. Higher levels of inflammatory markers have already been proven to be related to a decreased probability of venous recanalization [49]. Thus, the local hemodynamic conditions of venous valvular reflux, thrombus resolution, and multiple biomarkers (ICAM-1, P-Selectin, and NETs) are involved in the process of thromboembolism and PTS [46,50]. Based on these theories, the process of venous recanalization can influence the prevention of PTS through multiple pathways, including inflammatory effects. To demonstrate, Aleksandrov et al. [51] found a notable increase of inflammatory cytokines on the skin explants of warfarin-treated rats, proving that anticoagulants exhibiting effective anti-inflammatory properties, other than VKAs, could be helpful in terms of PTS prophylaxis. Due to glycan chains, heparin and LMWHs possess potent anti-inflammatory effects via the tissue factor pathway inhibitor (TFPI). The TFPI further inhibits the release of P-selectin and chemokines. Thus, the proliferation of lymphocytes and fibroblast endothelial proliferation are also blocked [52]. In summation, an elevated inflammatory marker indicated subsequent PTS and an anticoagulant with significant anti-inflammatory quality could be beneficial during the preliminary months of treatments.

Based on the abovementioned investigations, LMWH has been proven effective for DVT and subsequent PTS. It has a long history of clinical practice. In comparison to VKA, the evidence from clinical data favored LMWH for the prevention of PTS. However, the comparison of these data is difficult, since different methods for PTS diagnosis were used. In the Home-LITE study, Hull et al. [53] compared the risks of PTS and leg ulcers in a tinzaparin group and a usual care group, and suggested that there was a lower incidence of PTS after tinzaparin. However, unlike the common assessment, using the Villalta scale, the patients in the Home-LITE study answered eight questions regarding their symptoms (swelling, difficulty walking, discoloration, heavy sensation, enlarged vein, skin warmth and redness, worsening end-of-the-day swelling, and a need for compression stockings) for assessment. Alternatively, venographic evidence of an improved recanalization could prove the efficacy of LMWH, as supported by other studies as well [54,55]. A prospective study performed by González-Fajardo et al. [29] had a long-term follow-up to compare the prophylactic effect between enoxaparin and coumarin. The clinical results support the effectiveness of enoxaparin, since a lower incidence of PTS was observed in the group that used enoxaparin (60.7%) compared to the group treated with coumarin (70.5%). A systematic review conducted by Hull et al. [56] pooled nine studies and compared recanalization after LMWH and oral anticoagulants. After analysis, the overall OR was 0.77, which favored LMWH in terms of PTS prophylaxis. All the abovementioned clinical trials and reviews demonstrated better prophylaxis after treatment with LMWH. However, evidence from only a direct comparison between LMWH and VKA is not enough to reach a comprehensive conclusion, especially after the later introduction of DOACs and adjuvant therapies.

Statins (HMG-CoA reductase inhibitors) are a kind of traditional hypolipidemic medication. They are extensively used in the primary and secondary prevention of arteriosclerotic cardiovascular disease. Additionally, evidence from preclinical and clinical studies has suggested its efficacy in reducing the risk of venous thromboembolism (VTE) [57,58,59]. After the establishment of the murine venous thrombosis model, Kessinger et al. [57] proved that the venous thrombosis burden can be reduced after a daily treatment with atorvastatin or rosuvastatin due to the interaction between multiple cell types and biomarkers (neutrophils, macrophages, IL-6, PAI-1, NETs, etc.). He also demonstrated the anti-inflammatory property of statins via FDG-PET/CT [57]. With regard to statin’s application in clinical trials, the JUPITER trial indicated the potential role of rosuvastatin by comparing with a placebo group after an median 1.9-year follow-up [60]. Its anti-inflammatory property is beneficial for the treatment of venous thromboembolism. Thereafter, numerous studies on thrombosis-related topics have yielded encouraging outcomes. Recently, the SAVER study investigated the effect of rosuvastatin against the presence of PTS based on the Villalta scale [61]. However, this RCT failed to demonstrate the feasibility of reducing the Villalta score through the use of rosuvastatin. From our perspective, there were no exact description of standard care, which was given to patients in the control group. Even though the RCT has a high quality with a large enough sample size and proper analysis, we excluded this trial due to the difficulties in grouping according to treatment in the control group.

Four DOACs (dabigatran, apixaban, edoxaban, and rivaroxaban) have already been introduced in clinical practice to prevent VTE. A phase 3 trial proved that DOACs were at least comparable to VKA in dealing with recurrent VTE [62]. DOACs are safer due to their reduced risk of bleeding. Additionally, their advantages include fewer drug–food interactions, stable and predictable anticoagulant effects, and standard dosages. Unlike VKA, the periodical monitoring of coagulative function is no longer necessary for DOACs. Based on all these advantages, DOACs have gradually taken up the position of traditional anticoagulants and are now regarded as the first-line anticoagulants for patients with VTE [61]. There is still scarce scientific evidence to support the preventive effect of DOACs on PTS, which makes it difficult to select optimal anticoagulants from the various drugs [22]. Most of the DOAC-related studies compared the preventive effect on PTS between rivaroxaban and VKA, and the conclusions of all the included studies favored rivaroxaban against the occurrence of PTS. Recently, increasing interest has been given to other DOACs (dabigatran, apixaban, and edoxaban); so, we additionally collected data on PTS’s presence after patients were treated with these anticoagulants. With information from more involved studies, our NMA was able to suggest that edoxaban exerted a more positive role in PTS prevention than the other anticoagulants.

In subgroup analyses based on the severity of PTS, each SUCRA of rivaroxaban (0.89 in the mild/moderate group and 0.86 in the severe subgroup, respectively) was still higher than the other anticoagulants. The data on SUCRA indicated that rivaroxaban remains a reliable anticoagulant in preventing PTS. It is worth noting that an ongoing multicenter, open-label RCT (the TILE pilot study) comparing tinzaparin and rivaroxaban will provide more evidence of the effectiveness of both LMWH and rivaroxaban after its completion in October 2023.

Our meta-analysis is designed to combine information from several trials and provide evidence of the efficacy or validity of an intervention or treatment. Although the overall I^2^ of our Bayesian NMA model indicated minor heterogeneity, the I^2^ of the pairwise comparison between warfarin and dabigatran showed moderate heterogeneity (66.7%). This heterogeneity is mainly derived from the study performed by Wik et al. [38], partly because this is a cross-sectional study from the RE-COVER studies and only patients with pulmonary embolism (PE) were recruited [63,64]. PE is usually a life-threatening complication after the development of DVT. Since, obviously, these two diseases are closely related, in our study, patients who were affected by PE were not excluded for the sake of a better understanding of PTS prevention.

Edoxaban is a competitive inhibitor of factor Xa, which plays an important role in the amplification of the coagulation cascade. The Hokusai-VTE trial was conducted to compare edoxaban and dalteparin for the treatment of VTE, and it indicated that oral edoxaban has a non-inferior effect to dalteparin on VTE [65]. Regarding its efficacy and safety in atrial fibrillation, Marston et al. [66] demonstrated that edoxaban had better effectiveness than apixaban, dabigatran, rivaroxaban, and VKA in Germany. Lee et al. [67] found comparable effectiveness and safety when comparing edoxaban to rivaroxaban. All these abovementioned studies showed the efficacy of edoxaban against hypercoagulability. Interestingly, our study showed the potency of edoxaban on prophylaxis of PTS as well, which could probably be due to its inhibitory function on hypercoagulability.

Our Bayesian NMA provides strong and convincing evidence from the pooled data of seven anticoagulants. It also proves the efficacy of DOACs for PTS prevention. To our knowledge, this is the first NMA comparing multiple DOACs with traditional anticoagulants through both direct and indirect evidence simultaneously.

However, some limitations are still present: First, as this is a novel topic for PTS, there were limited clinical trials available, despite pooling 3812 cases. Second, the limited number of trials can potentially impact the study selection process and quality assessment. Therefore, we had to include all available observational and randomized studies. Third, the problem of heterogeneity should not be ignored, especially in the comparison of warfarin and dabigatran. Additionally, some confounders, such as the therapeutic schedule, duration of follow-up, doses, and adjunctive therapies, were not well-adjusted due to the limited number of studies. As shown in Table S6, the underestimating of heterogeneity may result from the absence of information regarding the length and type of DVT treatment.

## 5. Conclusions

With regard to its preventive effect on PTS, edoxaban appears to be an effective option against the occurrence of PTS via our assessment using the Villalta scale (Villalta score > 5). Edoxaban was then followed by LMWH + rosuvastatin, rivaroxaban, apixaban, LMWH, dabigatran, and warfarin. For mild/moderate and severe PTS, rivaroxaban continues to have a higher effectiveness than the other anticoagulants (warfarin, dabigatran, LMWH, and rivaroxaban). However, this conclusion should be elucidated with caution prior to the completion of more large-sized RCTs with good quality.

## Data Availability

The data presented in this study are available in the column of the Villalta score and recurrent DVT in Supplementary Material (Table S2).

## Supplementary Materials

The following supporting information can be downloaded at https://www.mdpi.com/article/10.3390/jcm12237450/s1, Figure S1: flow diagram of PRISMA; Figure S2: trace plots and density plots of MCMC; Figure S3: Gelman–Rubin plots; Figure S4: forest plots for heterogeneity of all included studies; Figure S5: forest plots for heterogeneity of the subgroup (Villalta score 5 to 14); Figure S6: forest plots for heterogeneity of the subgroup (Villalta score ≥ 15, ulceration); Figure S7: forest plots for heterogeneity of the subgroup (recurrent DVT); Figure S8: Venn diagram of distribution of thrombus; Table S1: PRISMA checklist; Table S2: characteristics of the included studies; Table S3: overview of the baseline data from the included studies; Table S4: relative effects of the included anticoagulants; Table S5: sensitivity analysis; Table S6: summary of therapies prior to anticoagulant and therapeutic duration.
