# Supplementary material for: A Systematic Review and Bayesian Network Meta-Analysis on the Effect of Different Anticoagulants on the Prophylaxis of Post-Thrombotic Syndrome after Deep Venous Thrombosis
Source: J Clin Med. 2023 Nov 30;12(23):7450. doi: 10.3390/jcm12237450 (PMC10706867; doi:10.3390/jcm12237450)
Supplement: Supplementary file 1 [file jcm-12-07450-s001.zip › Table S2. Character.pdf]

Table S2. Characteristics of the Included Studies

Study	Study design	Anticoagulants	Primary outcome	Secondary outcome	Therapeutic schedule	Follow-up	Villalta score	Recurrence of DVT
González-Fajardo 2008	Prospect cohort study	Enoxaparin<br>Coumarin	Villalta score	Recurrent VTE	Coumarin: INR 2-3 for at least 3 months<br>Enoxaparin: Twice daily 40 mg for 1 week followed by once-daily enoxaparin 40 mg for 3 months	1, 2, 3, 6, 12 months and yearly to 5 years	Enoxaparin Coumarin<br>Villalta score < 5 22 13<br>Villalta score 5 to 14 23 18<br>Villalta score ≥ 15 or venous ulcer 11 13	Enoxaparin Coumarin<br>10 17
Spiezia 2022	Prospect cohort study	Dabigatran<br>Rivaroxaban<br>Endoxaban<br>Apixaban	Villalta score	NA	Anticoagulant treatment were based on international guidelines and physicians	6, 12, 24 and 36 months	Dabigatran Rivaroxaban Apixaban Edoxaban<br>Villalta score < 5 80 265 153 119<br>Villalta score≥ 5 22 68 41 21	Dabigatran Rivaroxaban Apixaban Edoxaban<br>NA NA NA NA
Wik 2021	Cross-sectional study	Dabigatran<br>Warfarin	PRV	Original Villalta score<br>Recurrent VTE<br>HRQoL<br>VEINES-QOL	5–7 days of LMWH or intravenous unfractionated heparin<br>Dabigatran: 150 mg BID for 6 months<br>Wafarin: INR 2-3 for 6 months	3 years	Dabigatran Warfarin<br>Villalta score < 5 66 77<br>Villalta score 5 to 14 68 82<br>30 22<br>Villalta score ≥ 15 Venous ulcer 9 5	Dabigatran Warfarin<br>34 38
Sebastian 2018*	Prospect cohort study	Rivaroxaban<br>Warfarin	Venous patency rate	Villalta score	Rivaroxaban and VKA followed by endovascular therapy and heparin for at least 3 months	3, 6, 12 months	Rivaroxaban Warfarin<br>Villalta score < 5 31 19<br>Villalta score 5 to 9 4 1<br>Villalta score 10 to14 1 0<br>Villalta score ≥ 15 or venous ulcer 0 0	Rivaroxaban Warfarin<br>7 6
Prandoni 2019†	Prospect cohort study	Rivaroxaban<br>Warfarin	RTV	Villalta score<br>Recurrent DVT	Patients received conventional dose of rivaroxaban according to instructions<br>VKAs: Duration of anticoagulation was 3 months	3 years	Rivaroxaban Warfarin<br>Villalta score < 5 184 571<br>Villalta score 5 to 9 44 267<br>Villalta score 10 to14 19 115<br>Villalta score ≥ 15 or venous ulcer 12 83	Rivaroxaban Warfarin<br>29 143
Cheung 2016‡	Post-hoc analysis	Rivaroxaban<br>Warfarin	Cumulative incidence of PTS	Severity of PTS<br>Recurrent VTE	Rivaroxaban: 15 mg twice daily for 3 weeks, followed by 20 mg once daily<br>Subcutaneous enoxaparin followed by a VKA (either warfarin or acenocoumarol:target INR2-3)	Median follow-up: 57 months	Rivaroxaban Warfarin<br>Villalta score < 5 116 104<br>Villalta score 5 to 14 40 60<br>Villalta score≥15 or venous ulcer 6 10	Rivaroxaban Warfarin<br>34 29
Jeraj 2017	Cross-sectional study	Rivaroxaban<br>Warfarin	Villalta score	Recurrent DVT	Rivaroxaban: 15 mg twice daily for 3 weeks and 20 mg once daily<br>Warfarin overlapping with dalteparin until INR reached 2 to 3	12 to 36 months	Rivaroxaban Warfarin<br>Villalta score < 5 46 20<br>Villalta score 5 to 9 15 15<br>Villalta score 10 to14 0 3<br>Villalta score≥15 or venous ulcer 0 1	Rivaroxaban Warfarin<br>4 3
Utne 2018	Cross-sectional study	Rivaroxaban<br>Warfarin	PRV	EQ-5D-3L<br>VEINES-QOL/Sym	At least 90 days of anticoagulants either with initial LMWH and Warfarin or rivaroxaban	24 ± 6 months or at least 3 months after recurrent DVT	Rivaroxaban Warfarin<br>Villalta score < 5 89 61<br>Villalta score 5 to 9 35 46<br>Villalta score 10 to14 28 26<br>Villalta score≥15 or venous ulcer 9 15	Rivaroxaban Warfarin<br>6 12
Ferreira 2020	Cross-sectional study	Rivaroxaban<br>Warfarin	Prevalence of PTS	RTV	Rivaroxaban and Warfarin were treated for at least 3 months	15 months for patients treated with Rivaroxaban<br>61 months for patients treated with Warfarin	Rivaroxaban Warfarin<br>Villalta score < 5 35 18<br>Villalta score 5 to 9 29 22<br>Villalta score 10 to14 3 8<br>Villalta score≥15 or venous ulcer 4 10	Rivaroxaban Warfarin<br>10 12

deAthayde 2019	RCT	Rivaroxaban<br>Warfarin	Incidence of PTS<br>Venous recanalization	Prevalence of pulmonary embolism Death Complications	Subcutaneous enoxaparin every 12 h or intravenous unfractionated heparin for at least 48 to 72 h<br>Rivaroxaban: 15mg twice daily for 21 days after initial dose and 20 mg once daily for 6 months<br>Warfarin: sufficient to maintain an INR of 2 to 3 for 6 months or extended anticoagulant therapy	1,3,6 and 12 months	 Villalta score < 5<br>Villalta score 5 to 9<br>Villalta score 10 to14<br>Villalta score≥15 or venous ulcer	Rivaroxaban<br>42<br>4<br>0<br>0	Warfarin<br>27<br>7<br>4<br>0	Rivaroxaban<br>10	Warfarin<br>33
Norberto 2016	RCT	Rosuvastatin +Bemiparin<br>Bemiparin	D-dimer level	CRP decrease levels<br>Venous recanalization<br>PTS incidence	Bemiparin: Subcutaneously every 24 hours accordingto body weight for 7days,follow by 3 months therapy<br>Rosuvastatin+Bemiparin: 10 or 5 mg/d Rosuvastatin+ Bemiparin according to body weight	3 months	 n<br>Villalta score < 5<br>Villalta score ≥5	Rosuvastatin+Bemipari<br><br>70<br>44	Bemiparin<br>60<br>56	Rosuvastatin+Bemiparin<br>NA	Bemiparin<br>NA

Abbriviations: VTE: venous thromboembolism; DVT: deep vein thrombosis; INR: international normalized ratio; PRV: patient-reported Villalta scale; RTV: residual vein thrombosis; PTS: post-thrombotic syndrome; CRP: C-reactive protein
* This is a subgroup analysis of patients dignosed with iliofemoral vein thrombosis, followed by catheter-based early thrombus removal and stent placement.
† The estimate is from the a prospective cohort study and a historical cohort from 2003 to 2009.
‡ Data of the investigator-initiated cohort study is from an open-label, randomized, event-driven, noninferiority study (EINSTEIN study).
