# Supplementary material for: A Systematic Review and Bayesian Network Meta-Analysis on the Effect of Different Anticoagulants on the Prophylaxis of Post-Thrombotic Syndrome after Deep Venous Thrombosis
Source: J Clin Med. 2023 Nov 30;12(23):7450. doi: 10.3390/jcm12237450 (PMC10706867; doi:10.3390/jcm12237450)
Supplement: Supplementary file 1 [file jcm-12-07450-s001.zip › Table S3. Baseline data.pdf]

Table S3. Overview of the baseline data from the included studies

Stduty	Sample size, n		Age, years		Male, n		BMI		Proximal DVT, n		Unprovoked DVT, n		Elastic stocking, n	
González-Fajardo 2008		100												
	Coumarin	44 (44)	Coumarin	54.3 ± 15.2	Coumarin	25 (44.6)	Coumarin*	NA	Coumarin	40 (90.9)	Coumarin	21 (47.7)	Coumarin	34 (77.3)
	Enoxaban	56 (56)	Enoxaban	59.8 ± 13.3	Enoxaban	25 (56.8)	Enoxaban*	NA	Enoxaban	51 (91.1)	Enoxaban	21 (37.5)	Enoxaban	41 (73.2)
Spiezia 2022		769												
	PTS	152 (19.8)	PTS	60 ±17.8	PTS	77 (50.7)	PTS	28.3± 4.7	PTS	110 (72.4)	PTS	71 (46.7)	PTS	NA
	No PTS	617 (80.2)	No PTS	NA	No PTS	276 (44.7)	No PTS	NA	No PTS	340 (55.1)	No PTS	289 (46.8)	No PTS	NA
Wik 2021		349												
	Dabigatran	166 (47.6)	Dabigatran	52.0 ±13.5	Dabigatran	103 (62.0)	Dabigatran	29.9 ± 6.4	Dabigatran	166 (100)	Dabigatran	NA	Dabigatran	NA
	Warfarin	183 (52.4)	Warfarin	53.3 ±13.6	Warfarin	112 (61.2)	Warfarin	30.1 ± 6.1	Warfarin	183 (100)	Warfarin	NA	Warfarin	NA
Sebastian 2018		111												
	Rivaroxaban	73 (65.8)	Rivaroxaban	49 ± 21	Rivaroxaban	31 (42.5)	Rivaroxaban	26.6 ± 5.9	Rivaroxaban	73 (100)	Rivaroxaban	23 (31.5)	Rivaroxaban	NA
	VKA	38 (34.2)	VKA	40 ± 18	VKA	10 (26.3)	VKA	25.0 ± 3.9	VKA	38 (100)	VKA	10 (26.3)	VKA	NA
Prandoni 2019		1295												
	Rivaroxaban	259 (20.0)	Rivaroxaban	65.0 ± 15.6	Rivaroxaban	147 (56.8)	Rivaroxaban†	NA	Rivaroxaban	259 (100)	Rivaroxaban	199 (76.8)	Rivaroxaban	181 (69.9)
	VKA	1036 (80.0)	VKA	60.2 ± 17.5	VKA	508 (49.0)	VKA†	NA	VKA	1036 (100)	VKA	494 (47.7)	VKA	727 (70.2)
Cheung 2016		336												
	Rivaroxaban	162 (48.2)	Rivaroxaban	57 ± 16	Rivaroxaban	91 (56.2)	Rivaroxaban	28 ± 5	Rivaroxaban	92 (56.8)	Rivaroxaban	101 (62.3)	Rivaroxaban	111 (68.5)
	VKA	174 (51.8)	VKA	58 ±16	VKA	106 (60.9)	VKA	28 ± 5	VKA	117 (67.2)	VKA	114 (65.5)	VKA	134 (77.0)
Jeraj 2017		100												
	Rivaroxaban	61 (61.0)	Rivaroxaban	59 (50-68)	Rivaroxaban	37 (60.7)	Rivaroxaban	28.7 (25.3-31.6)	Rivaroxaban	42 (68.8)	Rivaroxaban	37 (60.7)	Rivaroxaban	NA
	Warfarin	39 (39.0)	Warfarin	60 (51-70)	Warfarin	20 (51.3)	Warfarin	28.7 (24.3-31.1)	Warfarin	25 (64.1)	Warfarin	21 (53.8)	Warfarin	NA
Utne 2018		309												
	Rivaroxaban	161 (52.1)	Rivaroxaban	60 ± 14	Rivaroxaban	109 (67.7)	Rivaroxaban	28 ± 5	Rivaroxaban	101 (62.7)	Rivaroxaban	87 (54.0)	Rivaroxaban	95 (59.0)
	Warfarin	148 (47.9)	Warfarin	63 ± 14	Warfarin	84 (56.8)	Warfarin	28 ± 5	Warfarin	92 (62.2)	Warfarin	76 (51.3)	Warfarin	97 (65.5)
Ferreira 2020		129												
	Rivaroxaban	71 (55.0)	Rivaroxaban	42 (33-56)	Rivaroxaban	32 (45.1)	Rivaroxaban	27.3 (23.8-31.5)	Rivaroxaban	71 (100)	Rivaroxaban	34 (47.9)	Rivaroxaban	50 (70.4)
	Warfarin	58 (45.0)	Warfarin	44 (29.5-52)	Warfarin	9 (15.5)	Warfarin	29.3 (25.6-34.5)	Warfarin	58 (100)	Warfarin	22 (37.9)	Warfarin	23 (39.7)
deAthayde 2019		84												
	Rivaroxaban	46 (54.8)	Rivaroxaban	54.93 ± 3.08	Rivaroxaban	20 (43.5)	Rivaroxaban	NA	Rivaroxaban	46 (100)	Rivaroxaban	20 (43.5)	Rivaroxaban	38 (82.3)
	Warfarin	38 (45.2)	Warfarin	55.61 ± 2.3	Warfarin	21 (55.2)	Warfarin	NA	Warfarin	38 (100)	Warfarin	21 (55.3)	Warfarin	33 (87.5)
Norberto 2016		230												
	Rosuvastatin + Bemiparin	114 (49.6)	Rosuvastatin + Bemiparin	66.0	Rosuvastatin + Bemiparin	65 (57.4)	Rosuvastatin + Bemiparin	NA	Rosuvastatin + Bemiparin	NA	Rosuvastatin + Bemiparin	53 (46.5)	Rosuvastatin + Bemiparin	NA
	Bemiparin	116 (50.4)	Bemiparin	63.13	Bemiparin	58 (50.0)	Bemiparin	NA	Bemiparin	NA	Bemiparin	82 (70.7)	Bemiparin	NA

Abbriviations: BMI: venous thromboembolism; DVT: deep vein thrombosis; PTS: post-thrombotic syndrome; VKA: vitamin K antagonist

* Data of BMI are not available, while data of weight (kg) are 74.17 ±11.9 on Enoxaban and 76.7 ± 10.5 on Coumarin, respectively.

† The corresponding data of BMI were recorded as number of patients whose BMI was over 25.

Data presented as mean, mean ± standard deviation, median (interquartile range), or number (%).
