# Supplementary material for: A Systematic Review and Bayesian Network Meta-Analysis on the Effect of Different Anticoagulants on the Prophylaxis of Post-Thrombotic Syndrome after Deep Venous Thrombosis
Source: J Clin Med. 2023 Nov 30;12(23):7450. doi: 10.3390/jcm12237450 (PMC10706867; doi:10.3390/jcm12237450)
Supplement: Supplementary file 1 [file jcm-12-07450-s001.zip › Table S4. Relative effects of the included pairs.pdf]

Table S4. Relative effects of the included anticoagulants

Apixaban						
-0.28(-1.06, 0.57)	Dabigatran		-1.05 (-5.17, 3.14)	-0.67 (-3.78, 2.40)		-0.05 (-2.82, 2.92)
0.43 (-0.46, 1.33)	0.71 (-0.18, 1.53)	Edoxaban				
-0.004 (-1.34, 1.35)	0.28 (-0.97, 1.52)	-0.43 (-1.78, 0.95)	LMWH	0.38 (-2.88, 3.50)		-1.10 (-1.94, 4.05)
0.17 (-0.58, 0.99)	0.45 (-0.19, 1.05)	-0.26 (-1.07, 0.58)	0.17 (-0.99, 1.30)	Rivaroxaban		
0.39 (-1.18, 2.02)	0.68 (-0.84, 2.19)	-0.03 (-1.63, 1.60)	0.40 (-0.46, 1.26)	0.22 (-1.19, 1.67)	LMWH + Rosuvastatin	0.72 (-0.40, 1.89)
-0.44 (-1.25, 0.40)	-0.16 (-0.79, 0.42)	-0.87 (-1.72, -0.005)	-0.44 (-1.53, 0.63)	-0.61 (-0.96, -0.27)	-0.83 (-2.25, 0.53)	Warfarin

The upper table indicates the log odds ratios derived from subgroup of recurrence of DVT with corresponding 95% CIs.
The lower table indicates results of the log odds ratios from overall network model including all anticoagulants with corresponding 95% CIs.

Dabigatran	0.20 (-1.11, 1.49)	-0.34 (-1.20, 0.53)	0.20 (-0.60, 0.99)
1.10 (-0.79, 2.96)	LMWH	-0.53 (-1.61, 0.58)	-0.0005 (-1.04, 1.04)
1.27 (-0.05, 2.77)	0.18(-1.33, 1.82)	Rivaroxaban	0.53 (0.18, 0.87)
0.54 (-0.69, 1.77)	-0.56 (-1.97, 0.87)	-0.73 (-1.50, -0.11)	Warfarin

The lower table shows the log odds ratios of subgroup (severe PTS) and The upper table shows the log odds ratio of subgroup (mild/moderate PTS).
