# Supplementary material for: A Systematic Review and Bayesian Network Meta-Analysis on the Effect of Different Anticoagulants on the Prophylaxis of Post-Thrombotic Syndrome after Deep Venous Thrombosis
Source: J Clin Med. 2023 Nov 30;12(23):7450. doi: 10.3390/jcm12237450 (PMC10706867; doi:10.3390/jcm12237450)
Supplement: Supplementary file 1 [file jcm-12-07450-s001.zip › Table S5. Sensitivity analysis.pdf]

Table S5. sensitivity analysis

	Main analysis (Villalta score ≥ 5)		Sensitivity analysis	Excluding studies of high risk	Sensitivity analysis	Excluding studies of small sample size*
	SUCRA(%)	Rank	SUCRA(%)	Rank	SUCRA(%)	Rank
Apixaban	48.4	4	49.3	4	48.3	4
Dabigatran	25.4	6	29.3	5	24.6	6
Edoxaban	80.0	1	81.5	1	80.8	1
LMWH	46.3	5	56.6	3	46.3	5
Rivaroxaban	64.4	3	66.3	2	64.5	3
LMWH + Rosuvastatin	74.0	2	-	-	74.7	2
Warfarin	11.4	7	17.0	6	10.8	7
pD	19.8		15.5		17.2	
DIC	45.5		35.0		36.8	

* Sample size of individual group < 50
