# Supplementary material for: A Systematic Review and Bayesian Network Meta-Analysis on the Effect of Different Anticoagulants on the Prophylaxis of Post-Thrombotic Syndrome after Deep Venous Thrombosis
Source: J Clin Med. 2023 Nov 30;12(23):7450. doi: 10.3390/jcm12237450 (PMC10706867; doi:10.3390/jcm12237450)
Supplement: Supplementary file 1 [file jcm-12-07450-s001.zip › Table S6. Therapies prior to anticoagulants and therapeutic duration.pdf]

Table S6. Therapies prior to anticoagulants and therapeutic duration

Studies	Treatments prior to corresponding anticoagulants	Duration of treatment
Spiezia 2022	-	-
Wik 2021	Subcutaneous low molecular weight heparin (LMWH) or intravenous unfractionated heparin.	5 to 7 days.
Sebastian 2018	Endovascular thrombus removal therapy followed by stent replacement.	-
Prandoni 2019	Unfractionated or LMWH overlapped with and followed by VKA.	-
Cheung 2016	Subcutaneous enoxaparin.	-
Jeraj 2017	Rivaroxaban group: 15 mg rivaroxaban twice daily.<br>Warfarin group: dalteparin in weight-adjusted dose.	Rivaroxaban group: three weeks.<br>Warfarin group: initial 5 to 7 days.
Utne 2018	Initial LMWH and warfarin or rivaroxaban.	-
Ferreira 2020	Warfarin group: enoxaparin	-
de Athayde 2019	Patients were hospitalized and received initial anticoagulation with subcutaneous enoxaparin or intravenous unfractionated heparin.	At least 48 to 72 hours.
Norberto 2016	-	-
González-Fajardo 2008	Twice daily enoxaparin 40 mg.	1 week.
