# Supplementary figures and images for: A Systematic Review and Bayesian Network Meta-Analysis on the Effect of Different Anticoagulants on the Prophylaxis of Post-Thrombotic Syndrome after Deep Venous Thrombosis
Source: J Clin Med. 2023 Nov 30;12(23):7450. doi: 10.3390/jcm12237450 (PMC10706867; doi:10.3390/jcm12237450)

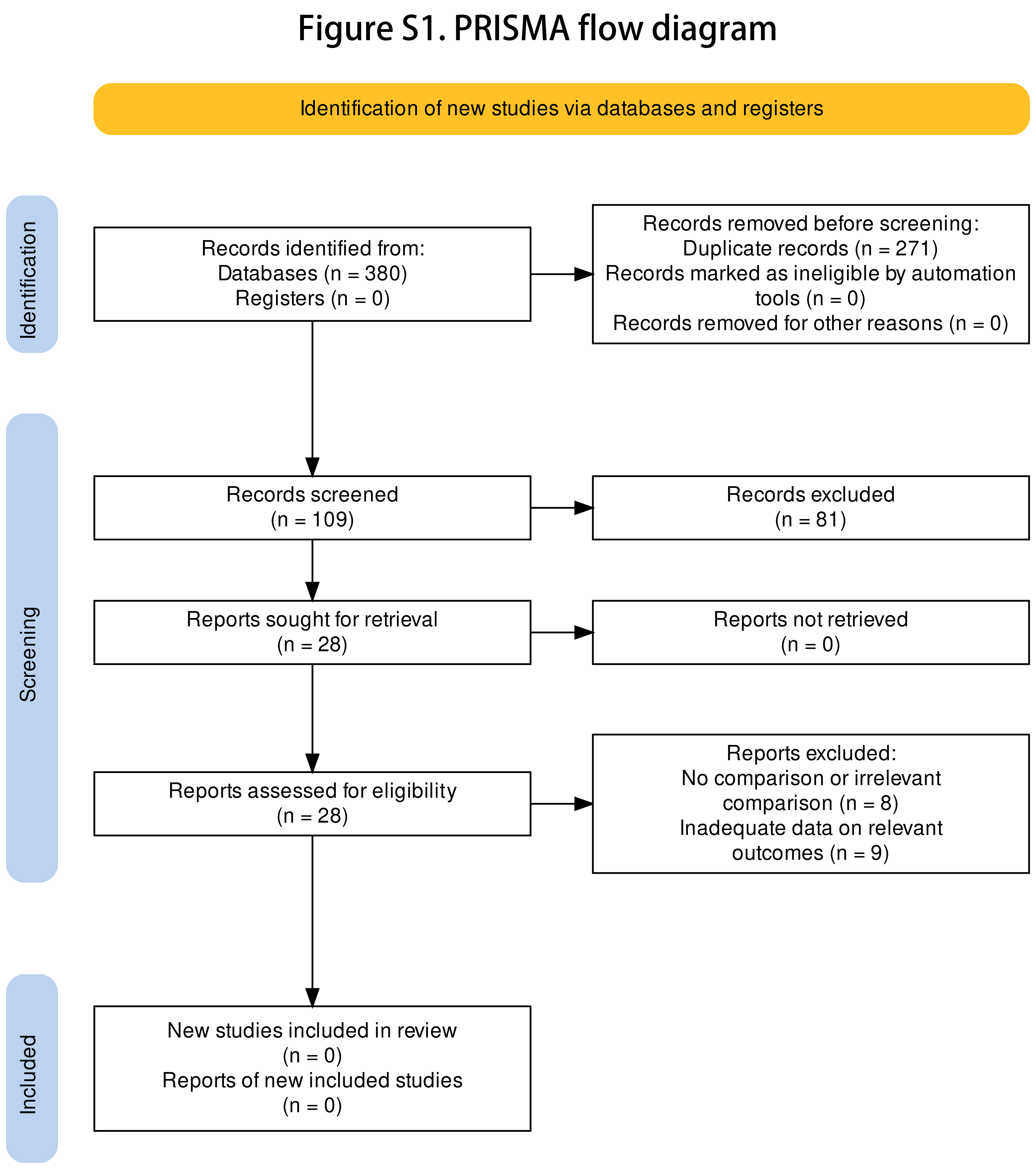

Supplement: Supplementary file 1 [file jcm-12-07450-s001.zip › Figure S1. Prisma.tif]

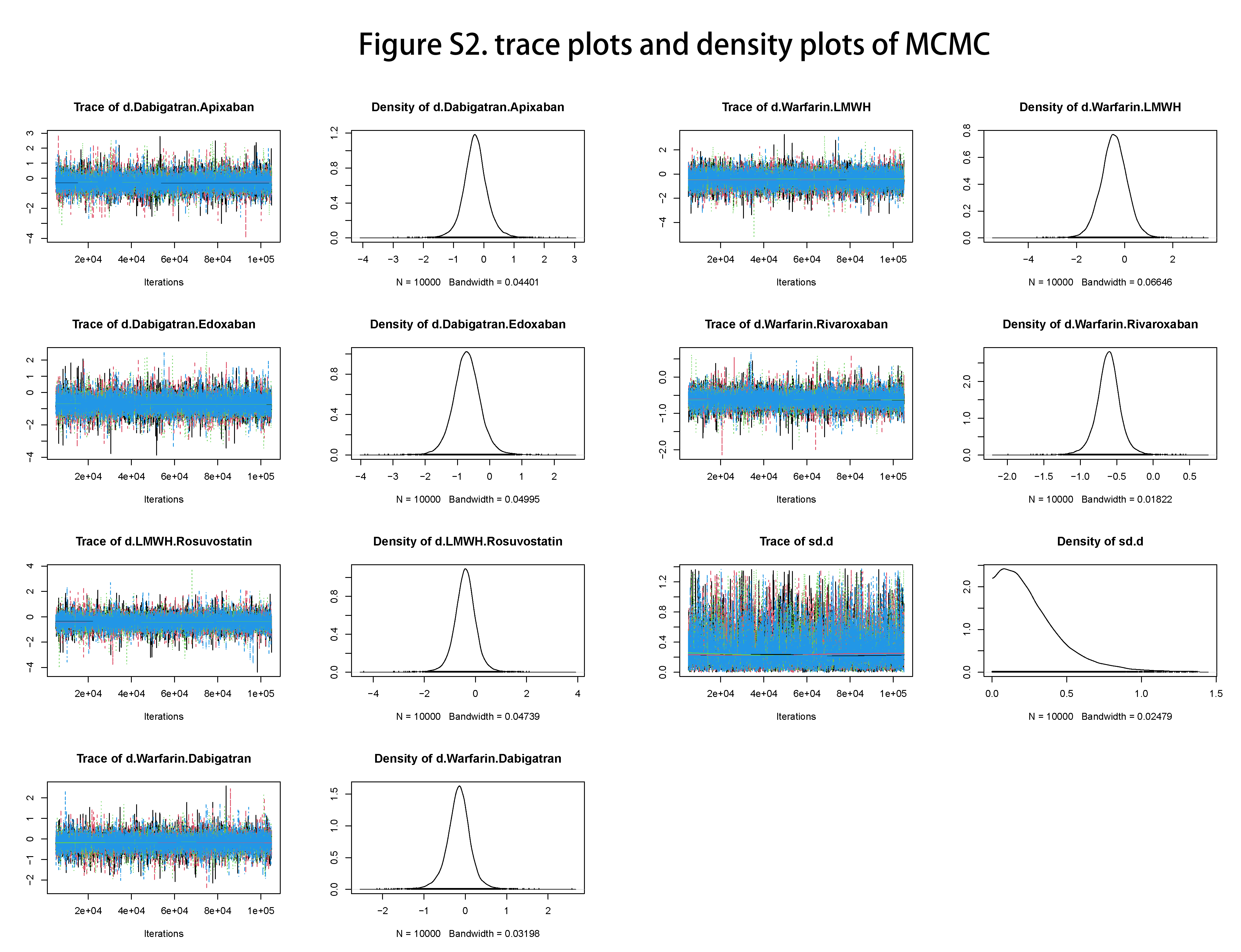

Supplement: Supplementary file 1 [file jcm-12-07450-s001.zip › Figure S2. Trace plots and density plots.tif]

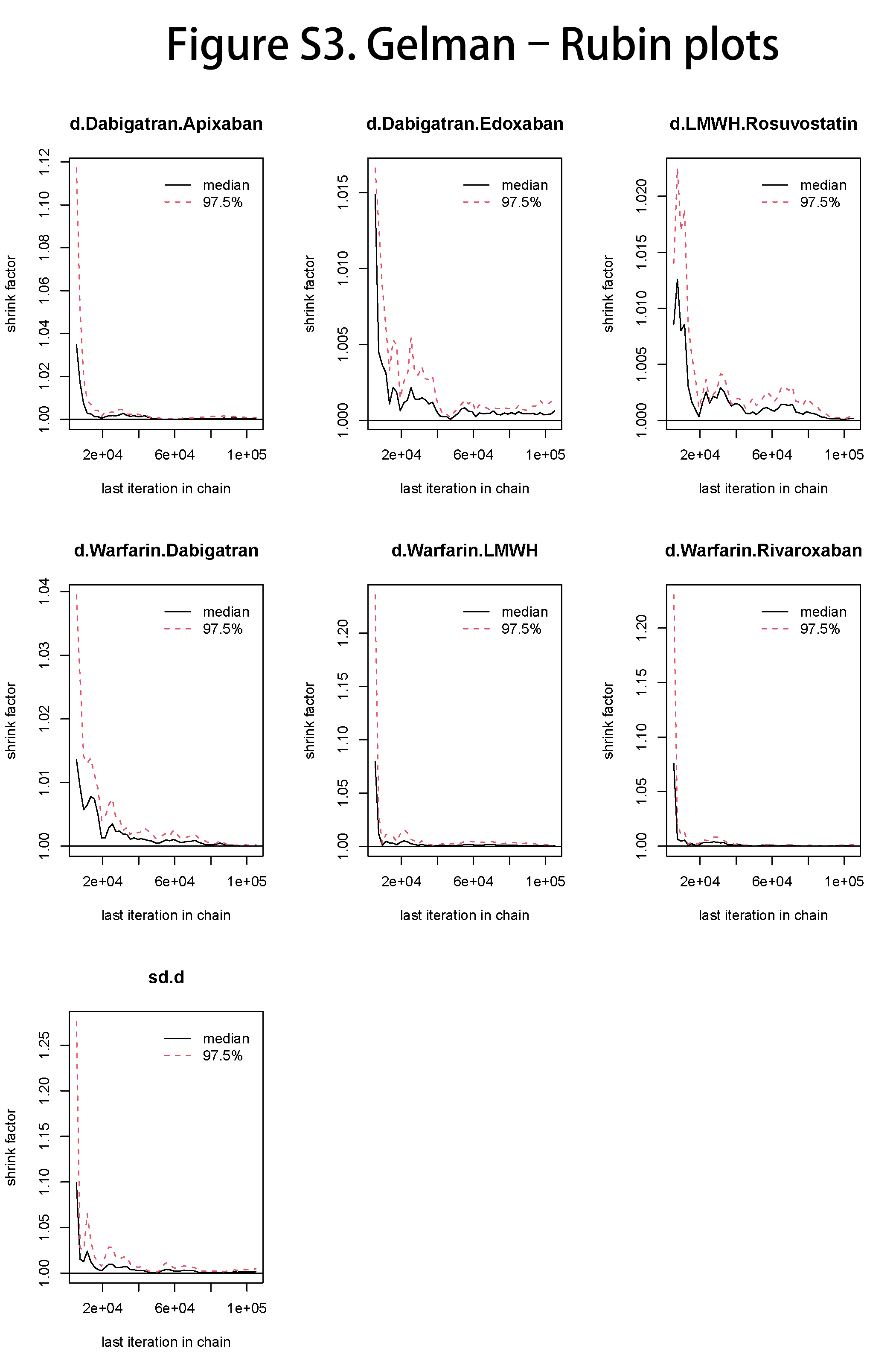

Supplement: Supplementary file 1 [file jcm-12-07450-s001.zip › Figure S3. Gelman plot.tif]

Forest plots for heterogeneity of all the included studies (Villalta score ≥ 5)

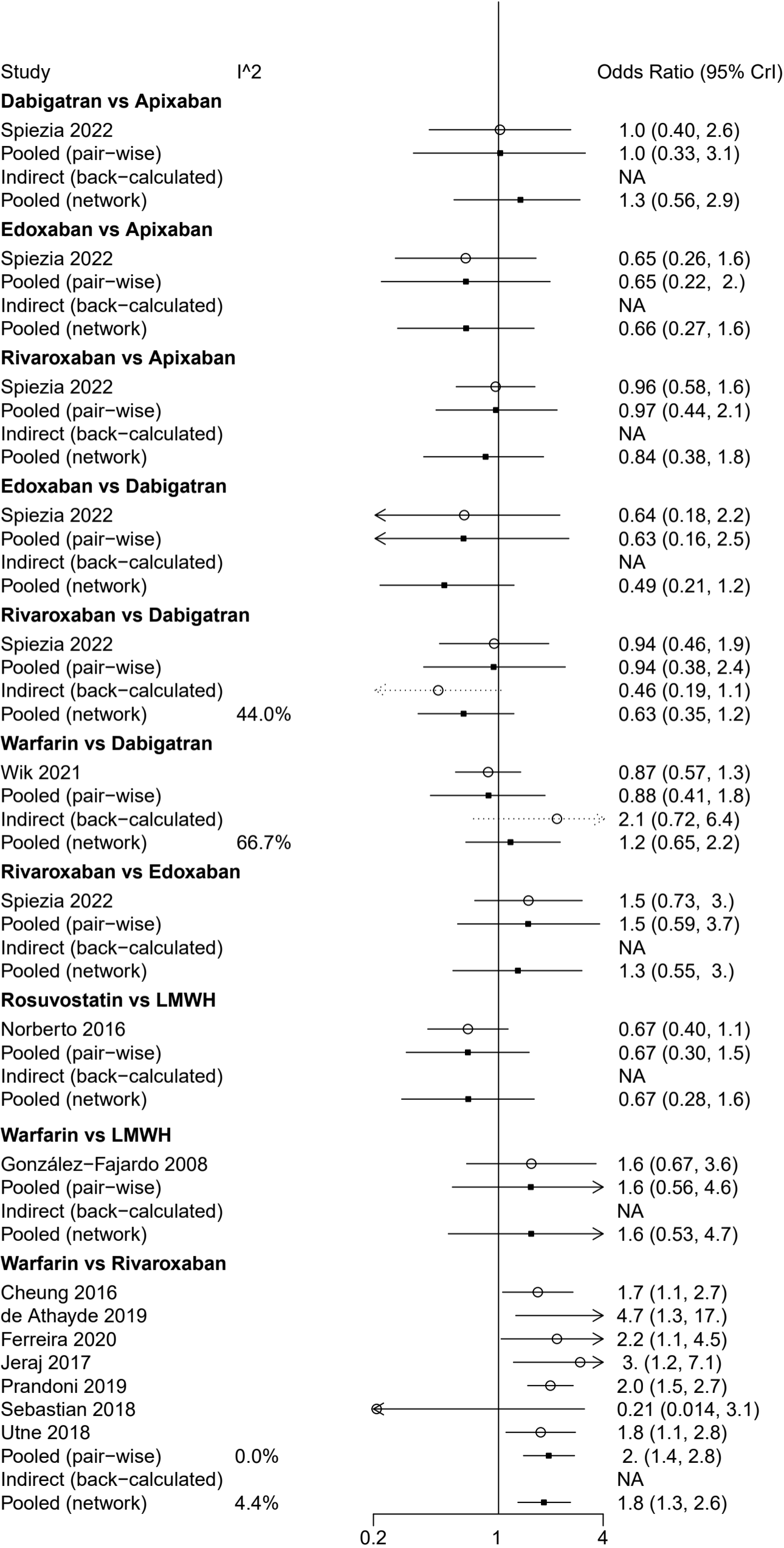

Supplement: Supplementary file 1 [file jcm-12-07450-s001.zip › Figure S4. Overall heterogeneity.pdf]

## Forest plots for heterogeneity of subgroup (Villalta score 5 to 14)

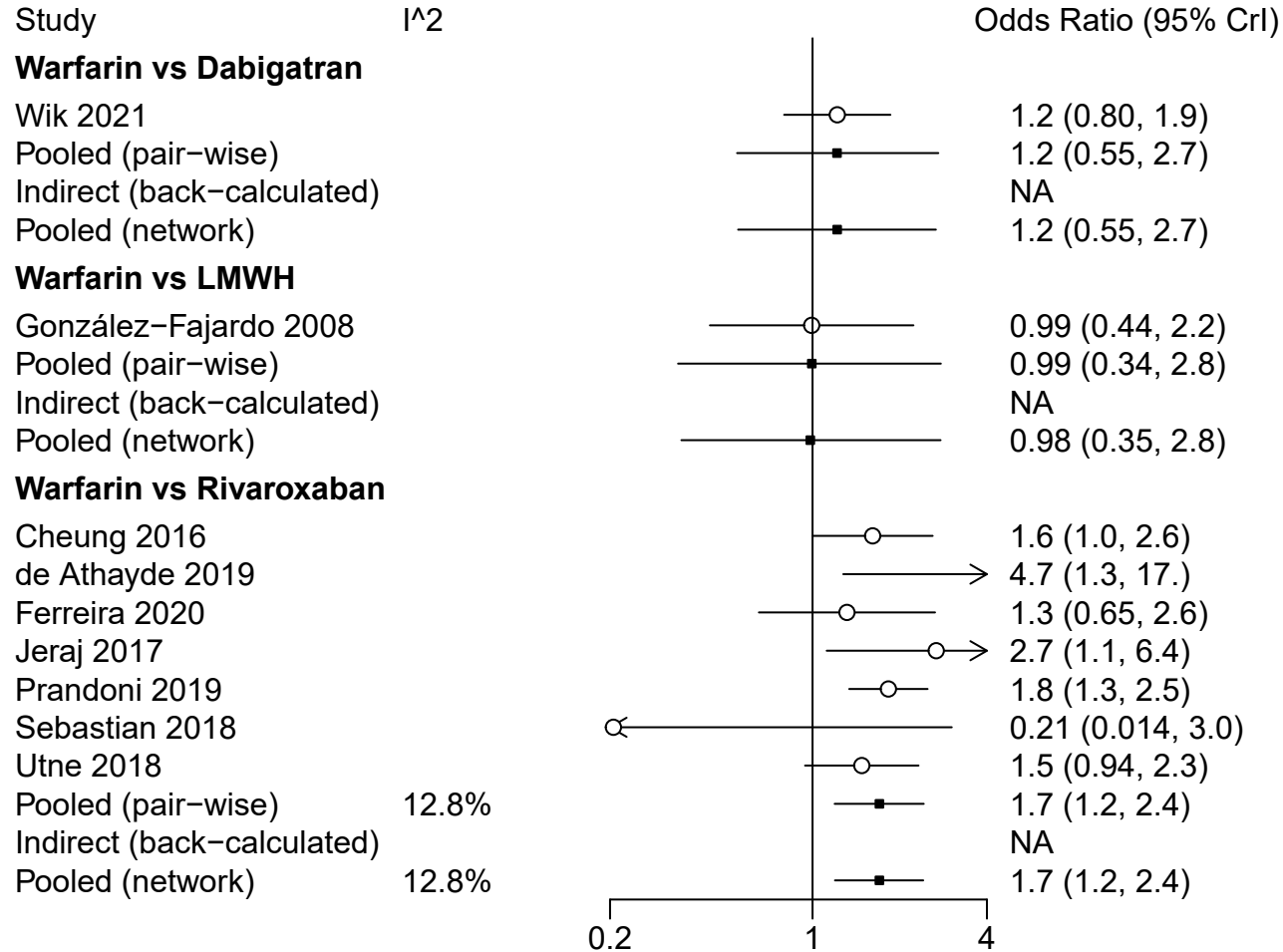

Supplement: Supplementary file 1 [file jcm-12-07450-s001.zip › Figure S5. Heterogeneity of mild and moderate PTS.pdf]

# Forest plots for heterogeneity of subgroup (Villalta score $\geq 15$ , ulceration)

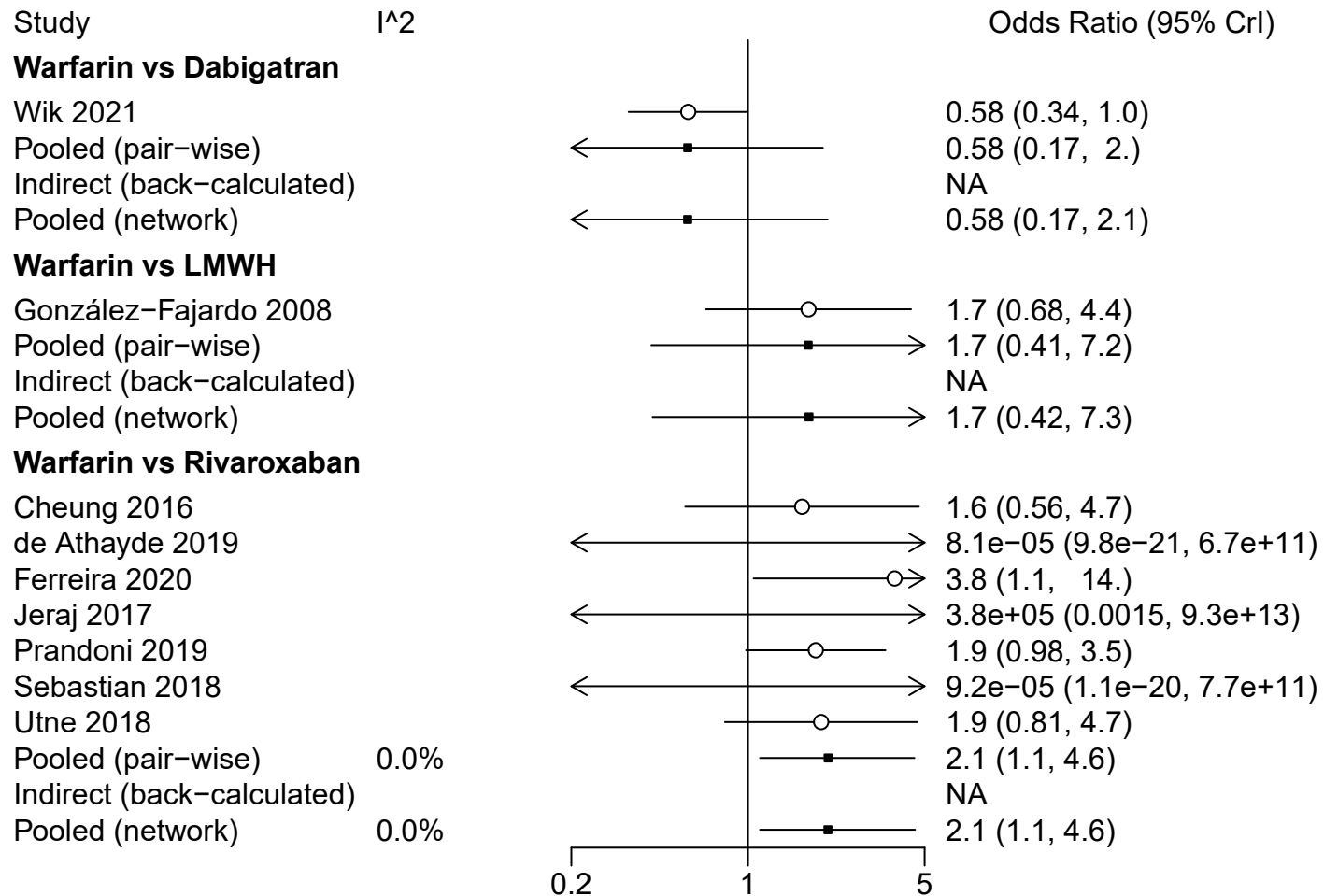

Supplement: Supplementary file 1 [file jcm-12-07450-s001.zip › Figure S6. Heterogeneity of severe PTS.pdf]

## Forest plots for heterogeneity of subgroup (recurrent DVT)

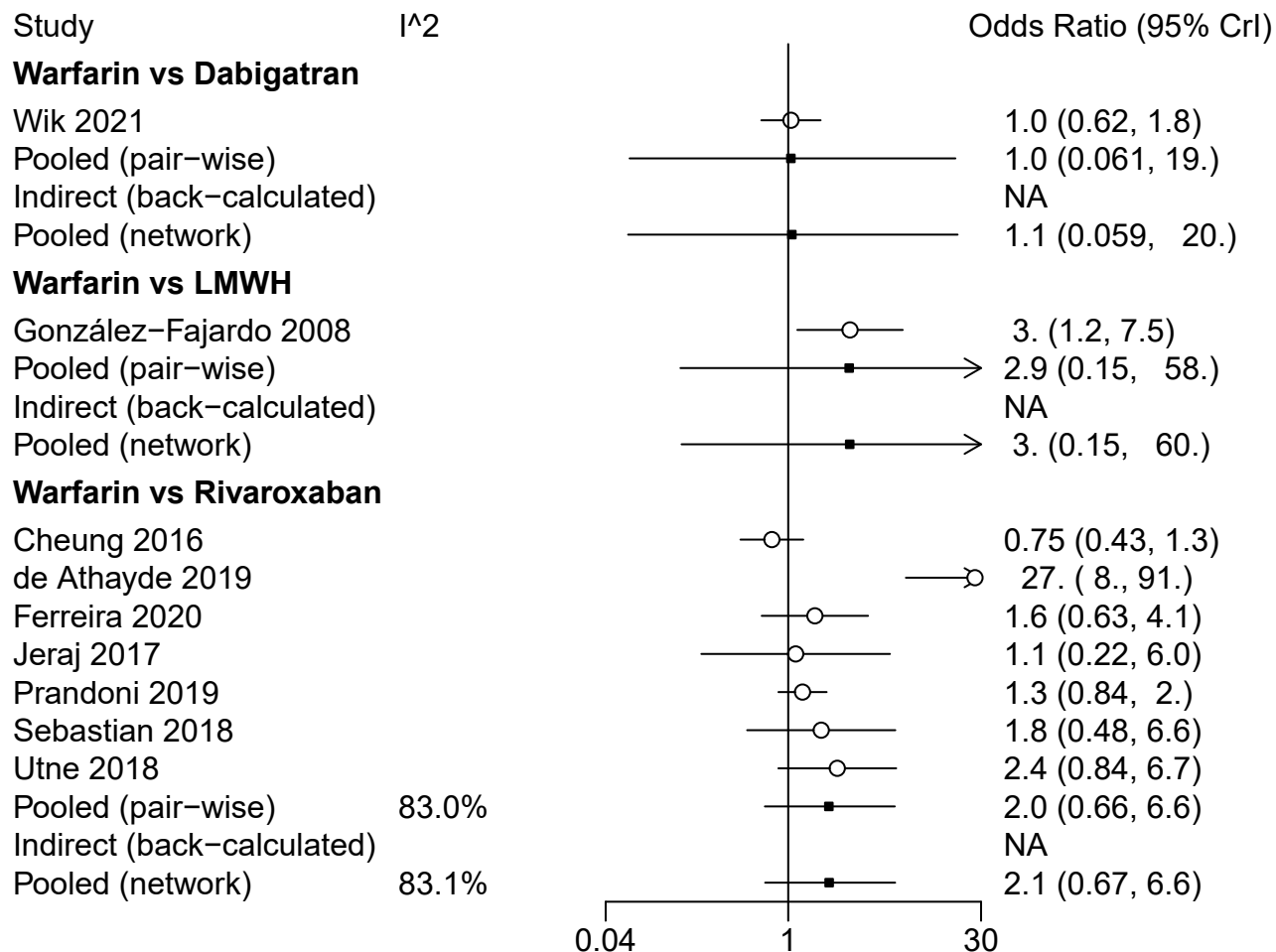

Supplement: Supplementary file 1 [file jcm-12-07450-s001.zip › Figure S7. Heterogeneity of recurrent DVT.pdf]

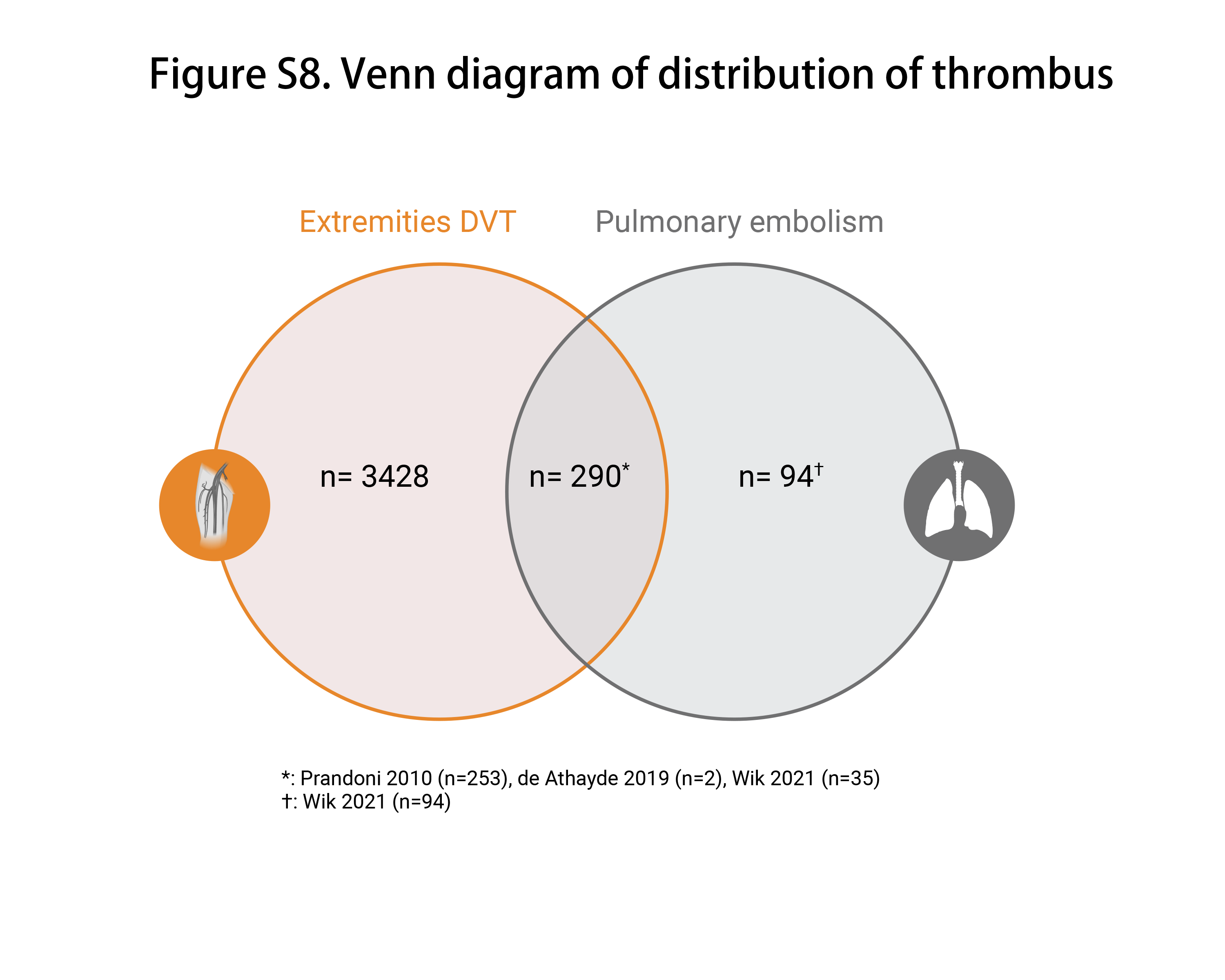

Supplement: Supplementary file 1 [file jcm-12-07450-s001.zip › Figure S8. Venn Diagram (distribution of thrombus).tif]
